# Prognostic impact of CDX2 in stage II colon cancer: results from two nationwide cohorts

**DOI:** 10.1038/s41416-018-0285-5

**Published:** 2018-11-14

**Authors:** Torben Frøstrup Hansen, Sanne Kjær-Frifeldt, Ann Christina Eriksen, Jan Lindebjerg, Lars Henrik Jensen, Flemming Brandt Sørensen, Anders Jakobsen

**Affiliations:** 1 Danish Colorectal Cancer Center South, Vejle Hospital, Vejle, Denmark; 2 Institute of Regional Health Research, University of Southern, Denmark, Odense, Denmark; 3 Danish Colorectal Cancer Group, Vejle, Denmark; 4 University Institute of Pathology, Aarhus University Hospital, and Department of Clinical Medicine, University of Aarhus, Aarhus, Denmark

## Abstract

**Background:**

The aim of the present study was to validate the prognostic impact of CDX2 in patients with stage II colon cancer.

**Methods:**

Two unbiased population-based cohorts representing all patients operated for stage II colon cancer in Denmark in 2002 and 2003. The CDX2 expression was evaluated by immunohistochemistry on whole tumour sections. Patients were classified into three groups, CDX2-positive, -moderate, and -negative, for comparison with the clinical data.

**Results:**

A total of 1157 patients were included. We found a significant relationship between loss of CDX2 expression and poor disease-free survival in both cohorts, *p* = 0.0267 and 0.0118, respectively. Five-year disease-free survival rates were 66%, 72% and 74% in the first cohort and 62%, 65%, and 75% in the second cohort for the negative, moderate, and positive CDX2 expression groups, respectively. Multiple Cox regression analysis performed on the combined cohorts confirmed an independent prognostic impact of CDX2 on disease-free survival, hazard ratio 1.543 (95% confidence interval 1.129–2.108), *p* = 0.0065.

**Conclusions:**

This retrospective study provides validation regarding the prognostic impact of CDX2 in patients with stage II colon cancer. The results justify prospective validation clarifying its clinical impact.

## Introduction

Most patients resected for stage I colon cancer are cured by the surgical procedure and all patients with stage III disease are offered adjuvant chemotherapy. The clinical challenge in localised colon cancer is the handling of patients with stage II disease due to the lack of simple and reliable prognostic biomarkers to identify patients at high risk of relapse.

The caudal-related homeobox transcription factor 2 (CDX2) is involved in intestinal cell development, differentiation, proliferation, and cell adhesion.^1,2^ It plays a crucial role during embryogenesis and later in life maintaining homeostasis of the intestinal epithelial cells. Loss of CDX2 has consequently been linked to increased cell migration and malignant transformation, often acting as a tumour suppressor in colon cancer, while in other settings related to an oncogenic role.^2^

Several studies have demonstrated an association between loss of CDX2 expression and clinical stage of colorectal cancer, which may be of prognostic importance, although contradictory results have been presented.^3–9^ Two studies have taken this hypothesis a step closer to the clinic. In 2016 Dalerba et al. published a study in *The New England Journal of Medicine* analysing the prognostic impact of CDX2 in a number of selected cohorts, including patients with stage II–III colon cancer. The rate of 5-year disease-free survival (DFS) was lower in patients with loss of CDX2 than in those with preserved CDX2-expressing tumours^10^ and the authors argued for further validation. Pilati et al.^11^ confirmed these results in a publication in *Annals of Oncology*, based on approximately 500 patients with stage II–III colon cancer, and called for final validation in large cohorts assessing CDX2 expression by immunohistochemistry.

In the present study we aimed to address this challenge by analysing the prognostic impact of CDX2 in two independent and unbiased, population-based cohorts of patients operated for stage II colon cancer.

## Materials and methods

This study is reported in accordance with REMARK.^12^

### Patient populations

We investigated two independent population-based cohorts retrieved by a search in the nationwide registry administered by the Danish Colorectal Cancer Group (DCCG). This database contains prospectively collected surgical and pathological data. The search identified all stage II colon cancer patients with tissue available from surgical treatment in 2002 (named first (test) cohort, *N* = 674) and 2003 (named second (validation) cohort, *N* = 668). Patients deceased within 60 days from the operation and those receiving adjuvant chemotherapy were excluded (*N* = 75 and 28 in the first cohort; *N* = 66 and 16 in the second cohort). All hospitals contributed to the first cohort (23 different departments) and only 2 of them were unable to participate in the second cohort. The final study cohort of 1157 patients thus represented 97% of the Danish population of stage II colon cancer in 2002 and 2003.

The national registry of all pathology reports in Denmark (*PatoBanken*) was used to identify patients with pathologically verified recurrence. Patients with other malignancies were also identified this way. To identify patients who had been in contact with a department of oncology with non-pathologically verified recurrence or who had died, the Cause of Death Registry and the National Patient Registry were consulted, respectively. Initiation of chemotherapy <90 days after the operation was marked as a case of adjuvant chemotherapy. A selected number of these cases (approximately 50%) were further validated by consulting the medical records in the respective departments of oncology.

Postoperatively, patients were followed by the treating department according to local guidelines. The patients were not routinely referred to a department of oncology.

The study was approved by the Regional Committee on Health Research Ethics and the Danish Data Protection Agency (test cohort S-20140119, 14/26345, and validation cohort S-20090049, 15/21683) according to Danish law. Prior to study start the Danish Registry of Human Tissue Utilisation was consulted.

### Sampling

Formalin-fixed, paraffin-embedded (FFPE) tissue blocks representing the deepest invasive front of the tumour of each patient were collected from the departments of pathology in Denmark. Thus, prior to inclusion, all histological slides from each case were screened by experienced pathologists (F.B.S. and J.L.) to retrieve the section with the deepest invasive front. Information on T-category, malignancy grade, neuronal and vascular invasion, and the number of lymph nodes assessed was obtained from the pathology reports. The term “not assessed” was used if the pathological feature was not described. Additional analysis of mismatch repair (MMR) status was performed in the case of missing data. Localisation and tumour perforation were available from the DCCG Registry and the surgeons’ reports.

### Immunostaining

#### CDX2 immunostaining

All FFPE tissue blocks were processed at the Department of Pathology, Vejle Hospital, Denmark. The immunostaining was performed using the Dako K8002 kit (Dako, Glostrup, Denmark). Tissue sections, 4-μm-thick, were mounted on (Dako K8020) FLEX IHC Microscope Slides and dried for 1 h at 60 °C. Deparaffinisation and antigen demasking were performed in EnVision FLEX Target Retrieval Solution, pH 9 at 97 °C for 20 min. Blocking of endogenous peroxidase was achieved by EnVision FLEX Peroxidase-Blocking Reagent for 5 min. Tissue sections were incubated for 30 min with an anti-CDX2 monoclonal mouse antibody (Dako M3636, clone DAK-CDX2) diluted 1:75 in Dako S2022 Antibody Diluent. The antibody signal was amplified through EnVision FLEX+, Mouse Linker for 20 min, and detection (secondary antibody) through the EnVision FLEX/HRP for 30 min. Washing in EnVision FLEX Wash Buffer was performed between each step. The diaminobenzidine (DAB) signal was amplified in 0.5% copper sulphate in Tris-buffered saline pH 7.6 for 10 min. The visualisation was accomplished using Dako’s EnVision FLEX DAB + Chromogen deluted in EnVision FLEX Substrate Buffer for 12 min. Dehydration was performed in graded alcohol solutions (70–99%). All sections were counterstained with Mayer haematoxylin. Negative controls, provided by leaving out the primary antibody, followed each staining batch, which all contained CDX2-stained cases serving as positive control of the CDX2 immunohistochemical stain.

#### MMR staining

Followed the same principle as presented for the CDX2 immunostaining. Staining was performed using monoclonal mouse antibodies: MLH1 (Novocastra, Leica, (Wetzlar) Germany, clone ES05, dilution 1:100, product code NCL-L-MLH1); MSH2 (Novocastra, Leica, clone 25D12, dilution 1:100, product code NCL-L-MSH2); MSH6 (BD Transduction Laboratories, clone 44/MSH6, dilution 1:200, material number 610919); and PMS2 (BD Pharmingen, clone A16–4, dilution 1:500, material number 556415).

### Evaluation of the immunostains

**Fig. 1:**
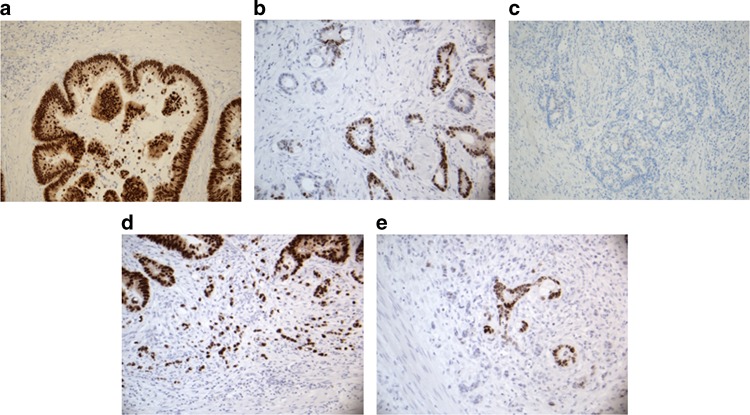
Examples of CDX2 expression. **a** Positive nuclear CDX2 expression in all tumour cells, **b** moderate CDX2 expression, **c** negative CDX2 expression, **d** budding cells with preserved expression of CDX2 and **e** budding cells with lost expression of CDX2

In brief, the section with the deepest invasive part of the adenocarcinoma included both the peripheral and central parts of the tumour. The slides were investigated morphologically at ×40 and ×100, and the subjective scoring of CDX2 expression was performed at ×200. Thus, within the whole tumour section the scoring of the CDX2 expression was performed according to Dalerba et al.,^10^ modified according to the results of our prior pilot study (*N* = 50 randomly selected cases) testing for the reproducibility of the scoring of CDX2 among two experienced pathologists (F.B.S. and JL). Tumours with nuclear CDX2 expression in all cancer cells were scored as positive. All adenocarcinomas completely lacking CDX2 expression or only showing nuclear expression in a minority of the cancer cells (≤50%) were scored as negative. Moderate expression applied to tumours with nuclear CDX2 expression above 50 and below 100%. Only nuclear CDX2 stain was considered as positive, and only invasive tumour areas were scored, excluding remnants of adenoma in the whole tumour section, and only tumour cells with obvious blue nuclear staining (Mayer’s haematoxylin) without any brown CDX2 reaction were scored as negative. Thus, in accordance with earlier published recommendations,^13^ the intensity of the immunohistochemical CDX2 stain was not taken into consideration, leaving the percentage of CDX2-positive adenocacrnoma cells as the sole parameter for scoring. Using this approach for CDX2 scoring of the 50 cases in the pilot series provided a kappa-value of 0.70 (weighted kappa-value = 0.67), whereas the strict use of the scoring scheme formulated by Dalerba et al.^10^ in our hands, produced a kappa*-*value of only 0.54. Representative examples of the three groups are provided in Fig. 1a–c. Our study group has interest in the tumour micro-environment, and special attention was thus displayed in detecting focal areas of CDX2-negative tumour budding cells in otherwise CDX2-positive or CDX2-moderate tumours. This quality was monitored in all cases morphologically evaluated to show budding, irrespective of the degree of tumour budding and the extension of the focal CDX2-negative tumour budding area (Fig. 1d, e). All tissue sections were scored for CDX2 expression by one experienced pathologist (F.B.S.) without knowledge of patient outcome. Assessment of MMR status was carried out by a supervised, training pathologist (A.C.E.). A tumour was classified as MMR-proficient (pMMR) if a positive nuclear staining was present for all four MMR proteins (MLH1, MSH2, MSH6, and PMS2). Tumours with loss of nuclear expression for at least one MMR protein were classified as MMR-deficient (dMMR).

### Statistics

The endpoint DFS was defined as time from operation to colon cancer recurrence or death of any cause. Patients later diagnosed with another cancer were censored at the time of diagnosis (*N* = 48 and 36 in the test and validation cohorts, respectively). The reproducibility of CDX2 scoring was tested by kappa-statistics, and the relationship between CDX2 expression and outcome measures was evaluated using Kaplan-Meier curves and compared by log-rank tests. The hazard ratio of individual, potentially prognostic variables was estimated by simple Cox regression, and variables with significant *p*-values were included in the multiple Cox regression analysis. Schoenfeld residuals test versus time was used to test the proportional hazard assumption.

All statistical calculations were carried out using the NCSS statistical software (NCSS Statistical Software, Kaysville, UT 84037, USA, version 2007). *p*-Values < 0.05 were considered significant and all tests were two-sided.

## Results

### Patient characteristics

Patients were followed for 7 years in both cohorts. A total of 90 and 86 recurrences and 239 and 270 deaths were found in the test and validation cohorts, respectively.

Clinical and pathoanatomic characteristics are presented in Table 1. The two cohorts were comparable at several parameters, including the scoring of CDX2. Significantly more women, tumours with a high malignancy grade, perforations, and patients with 12 or more lymph nodes removed were seen in the validation cohort.

**Table 1:** Clinical and pathoanatomic characteristics, *N* = 1157

Parameter	Test cohort	Validation cohort	*p*-Value^b^
	*N* = 571 (%)	*N* = 586 (%)	
Gender			
Male	282 (49)	254 (43)	**0.0393**
Female	289 (51)	332 (57)	
Age, median 73			
>73	279 (49)	289 (49)	0.8768
≤73	292 (51)	297 (51)	
T-category			
T3	499 (87)	507 (87)	0.6599
T4	72 (13)	79 (13)	
Malignancy grade			
High^a^	70 (12)	116 (20)	**0.0005**
Medium + low	501 (88)	470 (80)	
Localisation			
Right	273 (48)	302 (52)	0.2052
Left	298 (52)	284 (48)	
Perforation			
Yes	17 (3)	51 (9)	**0.0001**
No	529 (93)	535 (91)	
NA	25 (4)	0 (0)	
Lymph nodes retrieved			
≥12	214 (37)	268 (46)	**0.0072**
<12	351 (61)	318 (54)	
NA	6 (1)	0 (0)	
Perineural invasion			
Yes	26 (5)	50 (9)	0.3312
No	358 (63)	535 (91)	
NA	187 (33)	1 (0)	
Vascular invasion			
Yes	43 (8)	69 (12)	0.4179
No	385 (67)	517 (88)	
NA	143 (25)	0 (0)	
MMR status			
dMMR	172 (30)	170 (29)	0.6785
pMMR	399 (70)	416 (71)	
CDX2			
Positive	346 (61)	379 (64)	0.1714
Moderate	159 (28)	157 (27)	
Negative	66 (12)	50 (9)	

Some percentages do not equal 100% due to rounding of data. Bold values are used for highlighting significant test results (*p* < 0.05)

*MMR* mismatch repair, *dMMR* mismatch repair-deficient, *pMMR* mismatch repair-proficient, *CDX2* caudal-related homeobox transcription factor 2

^a^Including mucinous and sigillocellular tumours

^b^Not available (NA), data not included in the analyses (Chi-square statistics)

The relationship between CDX2 expression and the clinicopathologic characteristics is presented in Table 2. Significant relationships between loss of CDX2 expression and high malignancy grade, right-sided tumours, and dMMR were validated (*p* < 0.0001).

**Table 2:** Relationship between clinicopathologic characteristics and CDX2 expression

	CDX2 expression			
Parameter	Test cohort (*N* = 571)		Validation cohort (*N* = 586)	
	Positive/moderate/negative	*p*-Value	Positive/moderate/negative	*p*-Value
Gender				
Male	180 (64)/77 (27)/25 (9)	0.1045	182 (72)/65 (26)/7 (3)	**<0.0001**
Female	166 (57)/82 (28)/41 (14)		197 (59)/92 (28)/43 (13)	
Age, median 73				
≥73	163 (58)/76 (27)/40 (14)	0.1262	174 (60)/87 (30)/28 (10)	0.0826
<73	183 (63)/83 (28)/26 (9)		205 (69)/70 (24)/22 (7)	
T-category				
T3	300 (60)/143 (29)/56 (11)	0.4794	337 (66)/134 (26)/36 (7)	**0.0039**
T4	46 (64)/16 (22)/10 (14)		42 (53)/23 (29)/14 (17)	
Malignancy grade				
High^a^	23 (33)/15 (21)/32 (46)	**<0.0001**	60 (52)/26 (22)/30 (26)	**<0.0001**
Medium + low	323 (64)/144 (29)/34 (7)		319 (68)/131 (28)/20 (4)	
Localisation				
Right	128 (47)/94 (34)/51 (19)	**<0.0001**	154 (51)/101 (33)/47 (16)	**<0.0001**
Left	218 (73)/65 (22)/15 (5)		225 (79)/56 (20)/3 (1)	
Perforation^b^				
Yes	8 (47)/6 (35)/3 (18)	0.4860	27 (53)/18 (35)/6 (12)	0.1845
No	321 (61)/150 (28)/58 (11)		352 (66)/139 (26)/44 (8)	
Lymph nodes removed^b^				
≥12	128 (60)/59 (28)/27 (13)	0.7496	170 (63)/69 (26)/29 (11)	0.1872
<12	216 (62)/98 (28)/37 (11)		209 (66)/88 (28)/21 (7)	
Perineural invasion^b^				
Yes	17 (65)/6 (23)/3 (12)	0.9559	30 (60)/17 (34)/3 (6)	0.4251
No	225 (63)/92 (26)/41 (11)		349 (65)/139 (26)/47 (9)	
Vascular invasion^b^				
Yes	22 (51)/13 (30)/8 (19)	0.1367	45 (65)/19 (28)/5 (7)	0.9179
No	246 (64)/101 (26)/38 (10)		334 (65)/138 (27)/45 (9)	
MMR status				
dMMR	55 (32)/63 (37)/54 (31)	**<0.0001**	67 (39)/63 (37)/40 (24)	**<0.0001**
pMMR	291 (73)/96 (24)/12 (3)		312 (75)/94 (23)/10 (2)	

Percentages are included in parentheses. Bold values are used for highlighting significant test results (p < 0.05)

*p*-Values are based on the chi-square test

Sum of the percentages do not always equal 100% due to rounding of data

*CDX2* caudal-related homeobox transcription factor 2, *MMR* mismatch repair, *dMMR* mismatch repair-deficient, *pMMR* mismatch repair-proficient

^a^Including mucinous and sigillocellular tumours

^b^The numbers differ from the overall sample size due to “not available” data for the marked parameters according to Table 1

### CDX2 immunostaining

The CDX2 expression was evaluable in all tissue sections. The majority of tumours displayed distinct nuclear expression, but predominant cytoplasmic staining with sparse nuclear staining was also detected. The frequency of CDX2-negative, moderate and positive tumours in both cohorts combined was 10%, 27%, and 63%, respectively. In a number of tumours pronounced heterogeneity was found between the CDX2 staining in the overall tumour and the budding tumour cells.

### CDX2 and prognoses

**Fig. 2:**
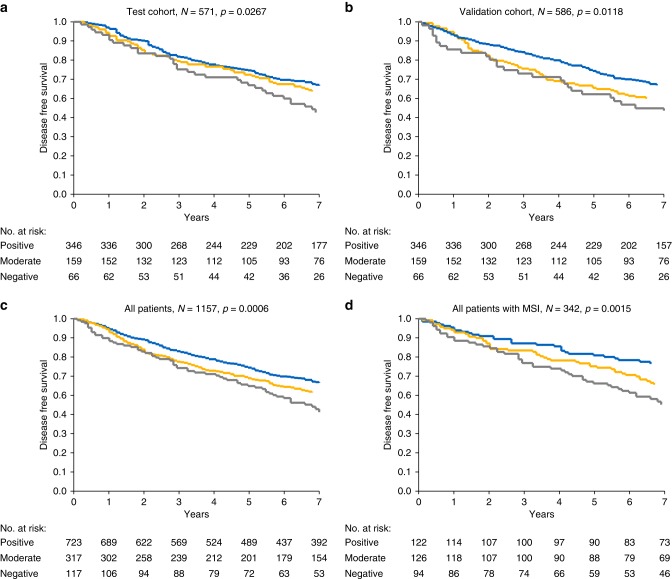
Disease-free survival curves. The test cohort (**a**), the validation cohort (**b**), the combined cohorts (**c**), and all patients with mismatch repair-deficient (dMMR/microsatelite instable (MSI)) tumours (**d**). Blue represents CDX2-positive, yellow CDX2-moderate, and grey CDX2-negative tumours

A significant relationship between loss of CDX2 expression and poor DFS was validated in both cohorts (Fig. 2a, b), *p* = 0.0267 and 0.0118, respectively. Five-year DFS rates were 66%, 72%, and 74% in the test cohort and 62%, 65%, and 75% in the validation cohort for the negative, moderate, and positive CDX2 expression groups, respectively. The combined cohorts are illustrated in Fig. 2c.

Table 3 shows the results from the Cox regression analysis with the two cohorts combined. Parameters showing significant impact in the simple analysis were included in the multiple analysis. An independent prognostic influence of CDX2 on DFS was confirmed with a hazard ratio of 1.543 (95% confidence interval 1.129–2.108), *p* = 0.0065.

**Table 3:** Cox regression analysis, disease-free survival, both cohorts (*n* = 953 in the multiple analysis)

Parameter	Simple analysis		Multiple analysis	
	HR (95% CI)	*p*-Value	HR (95% CI)	*p*-Value
Gender				
Male	1	**0.0471**	1	**<0.0001**
Female	0.829 (0.689–0.998)		0.645 (0.522–0.797)	
Age, median 73				
<73	1	**<0.0001**	1	**<0.0001**
≥73	2.041 (1.685–2.473)		2.150 (1.736–2.664)	
T-category				
T3	1	**<0.0001**	1	**<0.0001**
T4	2.074 (1.642–2.619)		1.819 (1.400–2.364)	
Malignancy grade				
Medium + low	1	0.2958		
High^a^	1.140 (0.892–1.457)			
Localisation				
Left	1	0.3694		
Right	0.919 (0.763–1.106)			
Perforation				
No	1	**<0.0001**	1	**<0.0001**
Yes	2.542 (1.854–3.484)		2.618 (1.834–3.677)	
Lymph nodes removed				
≥12	1	0.5163		
<12	1.065 (0.881–1.286)			
Perineural invasion				
No	1	**0.0072**	1	**0.0235**
Yes	1.568 (1.130–2.177)		1.506 (1.057–2.146)	
Vascular invasion				
No	1	**0.0191**	1	0.1287
Yes	1.414 (1.058–1.888)		1.278 (0.931–1.752)	
MMR status				
pMMR	1	0.1795		
dMMR	0.868 (0.706–1.067)			
CDX2				
Positive	1		1	
Moderate	1.226 (0.993–1.514)	0.0584	1.074 (0.847–1.362)	0.5575
Negative	1.682 (1.278–2.212)	**0.0002**	1.543 (1.129–2.108)	**0.0065**

Bold values are used for highlighting significant test results (p < 0.05)

*HR* hazard ratio, *CI* confidence interval, *MMR* mismatch repair, *dMMR* mismatch repair-deficient, *pMMR* mismatch repair-proficient, *CDX2* caudal-related homeobox transcription factor 2

^a^Including mucinous and sigillocellular tumours

### MMR deficiency and tumour budding

Two subgroups called for specific attention. Tumours with dMMR are generally considered to have a favourable prognosis, and adjuvant chemotherapy is questionable. The overall relationship between CDX2 expression and prognosis remained significant in patients with dMMR tumours (*N* = 342) as illustrated in Fig. 2d (*p* = 0.0015). The relationship between MMR status and CDX2 expression was primarily driven by lost expression of MLH1/PMS2.

**Fig. 3:**
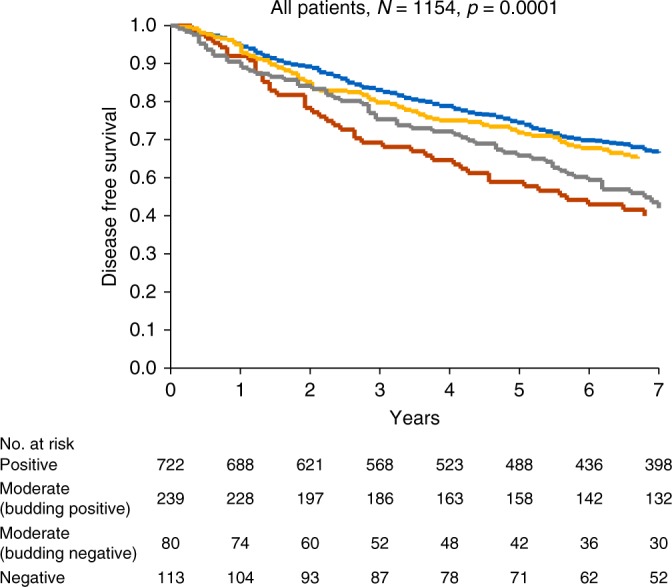
Disease-free survival curves for the combined cohort. Blue represents CDX2-positive, yellow CDX2-moderate with preserved expression of CDX2 in the budding cells, red CDX2-moderate with lost expression of CDX2 in the budding cells and grey CDX2-negative tumours

The clinical importance of tumour budding is often debated and therefore this subgroup was described as a separate entity. Focal loss of CDX2 expression in the budding tumour cells was detected in a subset of the patients (*N* = 80), with otherwise CDX2-positive or CDX2-moderate tumours (Fig. 1). This characteristic identified a subpopulation of patients with CDX2-moderate tumours having surprisingly poor prognoses, as illustrated in Fig. 3 (*p* = 0.0001). The 5-year DFS was only 60%. Loss of CDX2 expression in the budding cells was more frequently encountered in pMMR tumours 69/815 = 8% compared to microsatelite instable tumours 11/342 = 3%, *p* = 0.0013.

## Discussion

The present results from two population-based cohorts of stage II colon cancer patients confirm reduced expression of CDX2 to be related to a poor prognosis.

Loss of CDX2 expression was detected in 10% of the tumours, which is comparable to several previous studies.^5,6,10,11,14–16^ Differences may be related to the cut-off defining a CDX2-negative tumour and the investigated tumour specimens. Most studies are based on tissue microarrays, often with an unspecified core sampling procedure, which may hamper comparability, when addressing the invasive tumour front and the tumour micro-environment, including the budding tumour cells.^17,18^ A higher frequency of tumours with a high malignancy grade was seen in the validation cohort. This difference may partially be explained by the very subjective interpretation of this parameter. The increased frequency of perforation and patients with 12 or more lymph nodes removed is likely a consequence of the focus on the potential clinical importance of these parameters at the beginning of this millennium. These differences, however, do not influence the comparability of the two population cohorts, since sampling was unselected. The present study confirms previous results suggesting a relationship between loss of CDX2 expression and a higher malignancy grade, right-sided tumour, and dMMR.^3,4,6,7,11,15,19^ This is not surprising and a natural consequence of the relation to the consensus molecular subtype (CMS) group I as recently shown by Pilati et al.^11^

Being a nuclear immunostain, CDX2 is easy to evaluate, and highly reproducible, with reported kappa-values ranging between 0.85 and 0.97, depending on the number of scoring categories.^10^ Our scoring approach offered a kappa*-*value of 0.70, which is also suitable for clinical implementation. Thus, from the clinical point of view, immunohistochemical scoring of CDX2 is thus well-suited for diagnostic implementation.

The possible prognostic value of CDX2 has been assessed in several studies, often with contradictory results. This may be explained by methodological differences and perhaps more importantly, small heterogeneous single-centre cohorts and pooling of colon cancer patients representing all stages I–IV, which possibly also introduces treatment bias. In a recent, rather large study by Dalerba et al.^10^ the prognostic (and possible predictive) impact of CDX2 was analysed in a number of selected cohorts. In the two clinical cohorts 669 patients with stage II disease were assessed and lack of or low expression of CDX2 was detected in 7%. This group was also characterised by reduced 5-year DFS compared to the patients with CDX2-positive tumours.

The results from the current study on two large, nationwide population-based cohorts, unbiased regarding sampling and treatment, and addressing patients with stage II colon cancer only, confirm a prognostic impact of CDX2. Loss of expression is related to poor DFS. The impact remained significant after adjustment in a multiple Cox regression analysis combining the two cohorts. The present setup is ideal for testing prognostic markers since the unbiased cohorts represent the natural history of this disease. We decided to split the scoring of CDX2 into three categories, based on our prior pilot reproducibility study of the CDX2 scoring. Based on the current results, patients with negative CDX2 expression in their tumours seem to be the group of clinical interest, and the group with a moderate score should likely be handled in the same way as patients with CDX2-positive tumours. It is also important to emphasise that the current results cannot corroborate the hypothesis presented by Dalerba et al.,^10^ regarding the possible predictive value of CDX2, as this need testing in a different setting.

Patients with dMMR tumours constitute a specific group. They are often characterised by a favourable prognosis and adjuvant chemotherapy is not a standard according to the latest Danish guidelines. Based on the current results, however, reconsideration should perhaps be paid to patients with CDX2-negative tumours, since this group also demonstrated rather poor prognoses (5-year DFS of 68%). Another interesting result was associated with the tumour budding phenomenon. We noticed that tumours with positive or moderate CDX2 expression in general, showing focal loss of CDX2 expression in the budding cells in particular, reflected a surprisingly poor prognosis. Especially the subgroup with focal CDX2 loss of budding tumour cells in the CDX2-moderate group actually fared even worse than the CDX2-negative group with a 5-year DFS as low as 60%. This scenario likely reflects the initial changes related to the epithelial-mesenchymal transition. Since one of the main functions related to CDX2 is to maintain homeostasis in the epithelial layer, it is plausible that loss of this function is related to a cellular phenotype with an increased invasive potential. This is supported by publications describing transient downregulation of CDX2 at the invasive tumour front / tumour buds followed by re-expression at the metastatic sites^17,18^ and a recent study demonstrating the additive prognostic impact when addressing the poorly differentiated clusters together with the main tumour.^20^ Together with an overrepresentation of CDX2-positive and CDX2-moderate expression in pMMR tumours, poor prognosis links these observations to the CMS4 group.^21^

One limitation working with large nationwide data sets is the less-stringent insight into the individual patient record. Moreover, one cannot exclude a sometimes questionable central registration of the cause of death. These obstacles are inherent to the retrospective nature of this study and are the main reason for using verifiable endpoints such as death and recurrence rather than cancer specific endpoints. As a natural consequence of the presented results, CDX2 should be considered for a prospective validation elucidating its clinical impact in the daily clinic. This should preferably be a phase III study including patients resected for stage II disease, and with negative CDX2 expression in the tumour, to +/− adjuvant chemotherapy.

In conclusion, decreased expression of CDX2 is related to an inferior prognosis in stage II colon cancer. This especially concerns the otherwise prognostically favourable group of patients with dMMR tumours. Also, tumours with focal loss of CDX2 in the budding cells may represent a unique group with highly invasive potential. The current results call for prospective validation.

## Data Availability

All data are presented within the manuscript.
